# Chronic Obstructive Pulmonary Disease and Type 2 Diabetes Mellitus: Complex Interactions and Clinical Implications

**DOI:** 10.3390/jcm14061809

**Published:** 2025-03-07

**Authors:** Lucreția Anghel, Anamaria Ciubară, Diana Patraș, Alexandru Bogdan Ciubară

**Affiliations:** 1 Saint Apostle Andrew Emergency County Clinical Hospital, 177 Brailei St., 800578 Galati, Romania; anghel_lucretia@yahoo.com (L.A.); patras.diana@yahoo.com (D.P.); 2 Faculty of Medicine and Pharmacy, Dunarea de Jos University of Galati, 35 AI Cuza St., 800010 Galati, Romania; anamburlea@yahoo.com; 3 Doctoral School Biomedicine Science, University Galati, 800008 Galati, Romania

**Keywords:** COPD, type 2 diabetes mellitus, inflammation, insulin resistance, comorbidities, clinical management

## Abstract

Chronic obstructive pulmonary disease (COPD) and type 2 diabetes mellitus (T2DM) are highly prevalent chronic conditions, frequently coexisting due to their shared pathophysiological mechanisms and risk factors. Epidemiological studies estimate that up to 30% of COPD patients have comorbid T2DM, contributing to worsened disease progression, more hospitalizations, and higher mortality rates. Systemic inflammation in COPD contributes to insulin resistance by increasing pro-inflammatory cytokines (TNF-α, IL-6, and CRP), which impair glucose metabolism and beta-cell function. Conversely, hyperglycemia in T2DM exacerbates oxidative stress, leading to endothelial dysfunction, reduced lung function, and impaired pulmonary repair mechanisms. A comprehensive narrative review was conducted to evaluate the interplay between COPD and T2DM, examining shared pathophysiological mechanisms, clinical consequences, and management strategies. The co-occurrence of COPD and T2DM accelerates disease development, elevates hospitalization rates, and deteriorates overall prognosis. Pharmacological interactions complicate illness treatment, requiring a multidisciplinary therapy strategy. Recent data underscore the need to integrate palliative care, facilitate shared decision-making, and provide psychological support to enhance patient outcomes. Efficient therapy of COPD-T2DM comorbidity necessitates a customized, interdisciplinary strategy that targets both respiratory and metabolic health. Preliminary prognostic dialogues, palliative care, and holistic lifestyle modifications can improve patient quality of life and clinical results.

## 1. Introduction

COPD and T2DM represent two of the most common chronic conditions globally, with each exerting a considerable influence on quality of life and overall life expectancy. Recent findings indicate a significant correlation between these conditions, with individuals suffering from COPD exhibiting an increased likelihood of developing T2DM and vice versa [1,2]. Several shared risk factors contribute to the co-occurrence and bidirectional impact of these diseases. Smoking is a well-established risk factor for both COPD and T2DM, contributing to systemic inflammation, oxidative stress, and insulin resistance. Cigarette smoke contains pro-inflammatory compounds that increase levels of tumor necrosis factor-alpha (TNF-α), interleukin-6 (IL-6), and C-reactive protein (CRP), all of which are implicated in chronic low-grade inflammation and impaired insulin signaling [3]. Nicotine exposure also induces lipolysis, increasing circulating free fatty acids (FFAs), which contribute to hepatic insulin resistance and beta-cell dysfunction. Furthermore, chronic exposure to smoking-induced oxidative stress leads to mitochondrial dysfunction in skeletal muscle, impairing glucose uptake and further exacerbating insulin resistance [1,4]. Physical inactivity is a major contributor to insulin resistance, muscle atrophy, and metabolic dysfunction in patients with COPD and T2DM. The chronic breathlessness and fatigue experienced by COPD patients lead to reduced physical activity, which results in loss of skeletal muscle mass, decreased glucose uptake, and diminished insulin sensitivity [5]. Skeletal muscle plays a crucial role in glucose homeostasis, and its deterioration significantly impairs the body’s ability to regulate blood sugar levels. Additionally, sedentary behavior exacerbates systemic inflammation, mitochondrial dysfunction, and lipid accumulation, all of which further increase the risk of metabolic complications in COPD-T2DM patients [6]. Encouraging structured exercise interventions, pulmonary rehabilitation programs, and resistance training can help mitigate these effects by preserving muscle mass and improving insulin sensitivity [7].

Corticosteroids are frequently prescribed for COPD exacerbations due to their potent anti-inflammatory effects. However, their prolonged use is associated with significant metabolic disturbances. Systemic corticosteroids, such as prednisone, are known to impair insulin sensitivity by promoting hepatic gluconeogenesis, increasing glucose production, and reducing peripheral glucose uptake [8]. This effect is dose-dependent, with higher cumulative doses leading to a greater risk of hyperglycemia, weight gain, and visceral adiposity.

Even inhaled corticosteroids (ICSs), while delivering lower systemic bioavailability, have been linked to deteriorations in glycemic control, showing dose-dependent effects. A meta-analysis found that patients using high-dose ICS (>1000 μg/day of fluticasone equivalent) had a 34% increased risk of developing diabetes or experiencing worsening glycemic control [9]. Mechanistically, corticosteroids exacerbate lipolysis, impair pancreatic beta-cell function, and disrupt insulin receptor signaling, further complicating glycemic regulation in COPD-T2DM patients [10].

Given these overlapping risk factors and their significant impact on disease progression, it is crucial to adopt integrated management strategies that address both pulmonary and metabolic health simultaneously.

The incidence of T2DM in people with COPD is markedly elevated compared to the general population. Epidemiological studies demonstrate that persons with COPD possess an increased risk of acquiring T2DM as a result of chronic inflammation, oxidative stress, and physical inactivity [11]. Moreover, T2DM may accelerate the course of COPD by compromising lung function, amplifying vulnerability to infections, and deteriorating overall prognosis.

Multiple shared pathophysiological processes underlie the association between COPD and T2DM. Chronic inflammation is crucial, since increased pro-inflammatory cytokines contribute to insulin resistance and pulmonary dysfunction. Moreover, hypoxia, a prevalent characteristic of advanced COPD, affects glucose metabolism, worsening hyperglycemia and heightening the risk of diabetes-related complications [5]. Corticosteroid therapy’s effect on glucose homeostasis complicates illness management, requiring a meticulous and personalized therapeutic strategy.

Due to the considerable load of these comorbidities, there is an immediate necessity for a multidisciplinary therapeutic approach that combines pulmonary and metabolic care. Alongside disease-modifying therapies, the early integration of palliative care should be considered for patients with advanced or progressing conditions. Palliative care offers symptom treatment, psychological support, and advance care planning, thus enhancing overall patient well-being. Recent studies highlight that prompt palliative care referrals—both during acute health decline and in earlier phases—can improve quality of life and maximize resource efficiency [12].

This review examines the intricate relationships between COPD and T2DM, emphasizing the pathophysiological causes, clinical implications, and approaches to therapy designed to enhance patient outcomes.

## 2. Methodology

This narrative review was executed following an extensive literature search of electronic databases such as PubMed, Scopus, and Web of Science. The investigation included a blend of restricted vocabulary (MeSH terms) and free-text keywords pertinent to chronic obstructive pulmonary disease (COPD), type 2 diabetes mellitus (T2DM), inflammation, metabolic dysregulation, and comorbidities.

The search strategy was organized utilizing the SPIDER (Sample, Phenomenon of Interest, Design, Evaluation, Research Type) framework to encompass qualitative studies pertinent to the psychosocial dimensions of COPD-T2DM comorbidity. The search was limited to peer-reviewed publications published over the previous 15 years (2010–2025) to guarantee the incorporation of new discoveries. English-language journal articles were prioritized, whereas duplicate records and papers without adequate methodological data were removed.

Two researchers separately evaluated titles and abstracts to find papers that fulfilled the qualifying criteria. The complete texts of possibly pertinent publications were obtained for additional assessment. Discrepancies in study selection were addressed by discussion with a third researcher.

We performed a comprehensive evaluation of the quality of the included studies to guarantee methodological rigor and dependability. The assessment utilized the SANRA (Scale for the Assessment of Narrative Review Articles) criteria, which evaluates elements like the rationale for the review, thoroughness of the literature search, and clarity of evidence synthesis presentation.

To ensure a comprehensive appraisal of the retrieved studies, we also utilized the Mixed Methods Appraisal Tool (MMAT), which allows for the systematic assessment of qualitative, quantitative, and mixed-methods studies. The MMAT was chosen due to its adaptability in evaluating studies with diverse methodologies. Each study was scored based on criteria related to research design, data collection, and validity of conclusions.

Furthermore, we validated our technique against recognized frameworks utilized in narrative literature synthesis, as detailed in Jiang et al. [13]. We evaluated elements like research design, bias risk, sample size, and statistical robustness to assess the trustworthiness of the included studies. To improve openness, we elaborate on our quality evaluation technique in Appendix A.1, Appendix A.2 and Appendix A.3, outlining the inclusion/exclusion criteria and our rationale for research selection.

## 3. Pathophysiological Mechanisms Linking COPD and T2DM

### 3.1. Systemic Inflammation and Oxidative Stress

Both COPD and T2DM are marked by persistent low-grade inflammation. Increased concentrations of pro-inflammatory cytokines, including tumor necrosis factor-alpha (TNF-α), interleukin-6 (IL-6), and C-reactive protein (CRP), are implicated in the development of insulin resistance and the deterioration of pulmonary function. Oxidative stress intensifies tissue damage, disrupting pulmonary and metabolic balance [14].

Inflammation plays a central role in the bidirectional relationship between COPD and T2DM. TNF-α (tumor necrosis factor-alpha) is a key cytokine that is elevated in both conditions, which is known to inhibit insulin receptor signaling by inducing the serine phosphorylation of insulin receptor substrate-1 (IRS-1), leading to reduced glucose uptake and systemic insulin resistance [15]. Additionally, TNF-α stimulates lipolysis, increasing the circulation of free fatty acids (FFAs), which further impair insulin sensitivity and promote hepatic gluconeogenesis, contributing to worsening glycemic control in COPD-T2DM patients [16].

IL-6 is another pro-inflammatory cytokine that is overexpressed in COPD and T2DM, leading to hepatic glucose overproduction and dyslipidemia. Chronic IL-6 activation has been linked to pancreatic beta-cell dysfunction and apoptosis, further impairing insulin secretion and increasing the risk of diabetes progression [17]. Additionally, IL-6 disrupts mitochondrial oxidative phosphorylation, increasing oxidative stress-induced cellular damage in both pulmonary and pancreatic tissues [18].

CRP, an acute-phase reactant that is elevated in systemic inflammation, is also associated with endothelial dysfunction and increased vascular stiffness, worsening cardiovascular complications in COPD-T2DM patients [19]. High CRP levels correlate with increased arterial inflammation, reduced nitric oxide bioavailability, and heightened risk of atherosclerosis, further compounding the already elevated cardiovascular risk in these patients [20].

Chronic inflammation in COPD results in an imbalance between pro-inflammatory and anti-inflammatory mediators, worsening insulin resistance. The continual activation of inflammatory pathways, such as nuclear factor-kappa B (NF-κB) and the NLRP3 inflammasome, exacerbates systemic metabolic dysfunction [21]. Likewise, oxidative stress, caused by elevated reactive oxygen species (ROS) generation, affects pancreatic beta cells, limiting insulin release and exacerbating hyperglycemia [22].

Alongside systemic inflammation, COPD is linked to increased circulation concentrations of advanced glycation end-products (AGEs), which intensify oxidative stress and lead to vascular and metabolic problems. AGEs engage with their receptors (RAGEs) to initiate pro-inflammatory pathways, exacerbating tissue injury in the lungs and pancreatic islets [23]. This mechanism highlights the interaction between COPD-related inflammation and the progress of T2DM.

### 3.2. Insulin Resistance and Metabolic Dysregulation

Insulin resistance, a characteristic of type 2 diabetes mellitus, is often present in individuals with chronic obstructive pulmonary disease. Chronic hypoxia, physical inactivity, and systemic inflammation are factors that exacerbate metabolic abnormalities, hence impairing glucose homeostasis [24]. COPD-associated muscle atrophy plays a critical role in metabolic dysfunction, particularly in patients with comorbid T2DM [25]. Skeletal muscle is the primary site for insulin-stimulated glucose uptake, and its loss leads to decreased glucose storage capacity, increased insulin resistance, and dysregulated energy metabolism [26].

Muscle wasting in COPD results from chronic systemic inflammation, oxidative stress, physical inactivity, and increased activity in protein degradation pathways. Elevated TNF-α and IL-6 levels contribute to muscle catabolism by activating the ubiquitin–proteasome pathway, leading to progressive muscle mass loss [27]. Additionally, chronic hypoxia in COPD further exacerbates muscle dysfunction by reducing mitochondrial efficiency and ATP production, impairing muscle contractility and endurance [28].

This loss of muscle mass significantly impacts glucose metabolism because fewer muscle fibers are available for glucose uptake, leading to elevated blood glucose levels and worsened insulin sensitivity [29]. Moreover, impaired mitochondrial function in muscle cells contributes to decreased fatty acid oxidation and increased lipid accumulation, further aggravating metabolic syndrome and diabetes progression [30].

The impairment of lipid metabolism in COPD exacerbates insulin resistance. Elevated free fatty acids and modified adipokine release from dysfunctional adipose tissue, characterized by diminished adiponectin and heightened leptin, hinder glucose absorption in skeletal muscle [31]. The dysregulation of the hypothalamic–pituitary–adrenal (HPA) axis in individuals with COPD exacerbates metabolic dysfunction, resulting in heightened gluconeogenesis and hyperglycemia [32].

Additionally, systemic insulin resistance in COPD patients correlates with endothelial dysfunction, leading to compromised vascular function and heightened cardiovascular risk. The interplay between hyperglycemia and endothelial dysfunction intensifies pulmonary vascular remodeling, heightening the risk of pulmonary hypertension, a recognized consequence in severe COPD patients [33].

### 3.3. Impact of Hypoxia

Hypoxia, a prevalent characteristic of severe COPD, directly influences glucose metabolism by increasing insulin resistance and modifying pancreatic beta-cell activity. Chronic hypoxia causes mitochondrial dysfunction, diminishing ATP generation and disrupting insulin signaling pathways [34]. Moreover, intermittent hypoxia, observed in COPD patients with concurrent sleep apnea, exacerbates metabolic dysregulation by stimulating the sympathetic nervous system and elevating catecholamine secretion [35].

Intermittent hypoxia (e.g., as seen in obstructive sleep apnea, a common comorbidity in COPD) induces repeated episodes of hypoxia-reoxygenation injury, leading to sympathetic nervous system activation, increased catecholamine release, and insulin resistance [35]. These repeated hypoxic episodes promote oxidative stress and systemic inflammation, impairing glucose metabolism and increasing the risk of metabolic syndrome and T2DM [36].

Conversely, chronic hypoxia, seen in advanced COPD with chronic respiratory failure, results in persistent mitochondrial dysfunction and decreased ATP production, reducing skeletal muscle insulin sensitivity [28]. Additionally, chronic hypoxia exacerbates pulmonary hypertension, vascular endothelial dysfunction, and dysregulated erythropoiesis, increasing the risk of cardiovascular complications, which are already more prevalent in COPD-T2DM patients [37].

**Figure 1:**
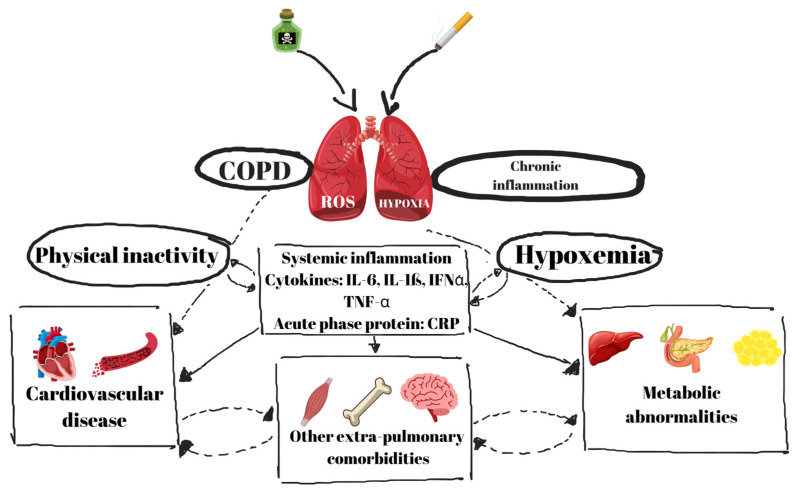
Pathophysiological mechanisms linking COPD and T2DM.

The metabolic effects of hypoxia influence hepatic glucose generation. Hypoxia-inducible factor-1 alpha (HIF-1α) is crucial for adaptation to hypoxic conditions; yet, its sustained activation in COPD has been associated with elevated hepatic gluconeogenesis and exacerbated hyperglycemia [38]. Hypoxia disrupts lipid metabolism, resulting in heightened lipolysis and the buildup of free fatty acids, which exacerbates insulin resistance. Furthermore, prolonged hypoxia is linked to increased oxidative stress, thus worsening inflammation and metabolic dysfunction in individuals with COPD and T2DM [39] (Figure 1).

### 3.4. Impact of Corticosteroids

The extensive utilization of systemic and inhaled corticosteroids in the therapy of COPD exacerbates hyperglycemia, hence complicating diabetic regulation. Corticosteroids enhance gluconeogenesis, disrupt insulin receptor signaling, and facilitate visceral fat formation, worsening metabolic disorders [9]. Prolonged corticosteroid medication is linked to a heightened risk of steroid-induced diabetes, necessitating vigilant monitoring in COPD patients (Table 1).

The impact of corticosteroids on muscle metabolism exacerbates sarcopenia, a disease marked by muscular atrophy and diminished physical function. Sarcopenia is common in COPD patients and exacerbates insulin resistance by reducing glucose absorption in skeletal muscle, resulting in compromised metabolic regulation in T2DM [40]. Extended corticosteroid use alters bone metabolism, elevating the risk of osteoporosis, which is especially alarming in individuals with both COPD and T2DM. Moreover, corticosteroids may inhibit endogenous cortisol synthesis, possibly resulting in adrenal insufficiency and additional metabolic instability [41].

**Table 1:** Impact of corticosteroid use on type 2 diabetes mellitus (T2DM) risk in COPD patients.

Study	Study Design	Patients	Age Range	COPD Severity	Corticosteroid Type	Duration of Use	Key Outcomes
Suissa et al. [42]	Case control	388,584	Not specified	Not specified	High dose (≥1000 μg/day fluticasone equivalent)	Median follow-up: 5.5 years	High-dose ICS use associated with a 34% increased risk of diabetes onset and progression.
Price et al. [43]	Matched cohort	17,970	≥40 years	All stages	Mean daily exposure ≥500 μg	Median follow-up: 5.3 years	Long-term ICS therapy associated with increased risk of diabetes onset and progression and osteoporosis.
Slatore et al. [44]	Observational cohort	50,148	Not specified	Not specified	Not specified	7 years	ICS use associated with a moderate dose-dependent increase in the occurrence of type 2 diabetes.
Faul et al. [9]	Randomized controlled trial	12	Not specified	Not specified	Not specified	6 weeks	No clinically significant change in HbA1c levels with ICS therapy in T2DM patients.
Boursi et al. [45]	Retrospective cohort	39,694	Not specified	Not specified	Not specified	Not specified	ICS use associated with a higher risk of diabetes onset, particularly at higher doses.
Marc et al. [46]	Systematic review and meta-analysis	38 trials	Not specified	Not specified	Not specified	Not specified	ICS use associated with a 21% increased risk of diabetes onset; higher doses linked to greater risk.
Tse et al. [47]	Observational study	58,955	Not specified	Not specified	Oral corticosteroids	6.9 years	Multiple adverse outcomes: type 2 diabetes mellitus, osteoporosis
		10 trials					
Kholis et al. [48]	Systematic review and meta-analysis		Not specified	Noy specified	High-dose ICS (>900 μg/day)	52-week follow-up	Significant increase in the risk of diabetes
Sttalberg et al. [44]	Observational study	7078	68.6 years	Not specified	High-dose ICS	Not specified	The risk of T2DM was 100%
Bazell et al. [49]	Retrospective study	Not specified	Not specified	Not specified	>1000 mg of prednisolone	48 months	Higher incidence of new conditions or events including cardiovascular disease, hypertension, obesity, type 2 diabetes

### 3.5. COPD-T2DM Comorbidity: Impact on Hospitalization, Mortality, and Complications

Several studies have shown that patients with both conditions experience the following:

- Higher hospitalization rates, with increased frequency and severity of COPD exacerbations due to impaired immune responses, chronic systemic inflammation, and oxidative stress [50]. The pro-inflammatory state caused by T2DM can lead to a greater risk of acute exacerbations of COPD (AECOPD), resulting in more frequent hospital admissions and prolonged hospital stays [51].
- Elevated mortality risk, as metabolic dysregulation worsens lung function decline and increases susceptibility to cardiovascular complications, such as heart failure, arrhythmias, and myocardial infarction [52]. Studies have reported that COPD patients with T2DM have a 30–50% increased risk of mortality compared to those without diabetes, largely due to accelerated endothelial dysfunction and heightened inflammatory responses [53].
- Greater incidence of complications, including heart failure, chronic kidney disease (CKD), and increased susceptibility to infections, such as pneumonia and sepsis [54]. Impaired immune function and altered inflammatory responses in COPD-T2DM patients lead to an increased burden of secondary infections, further complicating disease management [55].
- Patients with COPD-T2DM are also at a higher risk of prolonged hospital stays and readmissions, partly due to steroid-induced hyperglycemia and poor glycemic control during exacerbations [56]. The combination of hyperglycemia and inflammation has been shown to delay lung tissue repair, worsening post-exacerbation recovery [57]. Additionally, hyperglycemia impairs pulmonary microcirculation, leading to a reduction in oxygen delivery and the exacerbation of hypoxemia-related complications in COPD patients [24].
- T2DM contributes to vascular dysfunction, leading to impaired oxygen transport and worsened pulmonary hypertension in COPD patients [58]. Chronic hyperglycemia accelerates arterial stiffness and endothelial dysfunction, increasing the risk of coronary artery disease, cerebrovascular events, and sudden cardiac death in these patients [59]. Furthermore, diabetic nephropathy and CKD, common complications of T2DM, further increase the risk of volume overload and worsening heart failure, which is already a significant concern in COPD patients due to increased right ventricular strain and pulmonary hypertension [60].

## 4. Clinical Implications

### 4.1. Disease Progression and Exacerbations

The concurrent presence of COPD and T2DM correlates with elevated COPD exacerbations, extended hospitalizations, and increased death rates. Hyperglycemia diminishes immunological function, heightening vulnerability to respiratory infections and exacerbating pulmonary consequences [61]. Moreover, persistent systemic inflammation and oxidative stress accelerate the deterioration of lung function, resulting in a greater incidence of acute exacerbations and an elevated risk of respiratory failure [62]. Patients with COPD and uncontrolled diabetes demonstrate decreased responsiveness to bronchodilator medication and extended recovery durations after exacerbations, hence complicating disease management [63]. Preventive strategies include routine immunizations, enhanced glycemic management, and customized pulmonary rehabilitation programs, all of which are crucial for decreasing exacerbation rates and enhancing patient outcomes [64].

### 4.2. Cardiovascular Risk

The simultaneous development of COPD and T2DM markedly elevates the risk of cardiovascular disease (CVD), encompassing myocardial infarction, stroke, and heart failure [65].

Increased systemic inflammation, oxidative stress, and chronic hypoxia in COPD-T2DM promote cardiac remodeling, diastolic dysfunction, and left ventricular hypertrophy, ultimately predisposing patients to heart failure with preserved ejection fraction (HFpEF) [66]. Additionally, chronic hyperglycemia accelerates myocardial fibrosis and impairs myocardial relaxation, increasing the risk of heart failure with reduced ejection fraction (HFrEF) [67].

COPD and diabetes are both associated with autonomic neuropathy, characterized by increased sympathetic nervous system activation and reduced vagal tone. This dysregulation leads to higher heart rate variability, increased risk of atrial fibrillation, and ventricular arrhythmias, further compounding cardiovascular risks in COPD-T2DM patients [68].

Chronic lung disease leads to hypoxia-induced pulmonary vasoconstriction, which, when compounded by hyperglycemia-mediated vascular stiffness, contributes to pulmonary hypertension and cor pulmonale (right heart failure) [69]. The combination of chronic inflammation and metabolic dysregulation worsens right ventricular overload, leading to an increased risk of arrhythmias and sudden cardiac death [70].

Persistent hyperglycemia reduces NO bioavailability, leading to vasoconstriction, increased vascular permeability, and arterial stiffness. The result is a higher predisposition to hypertension, ischemic heart disease, and stroke [71]. Additionally, the activation of advanced glycation end products (AGEs) and their receptors (RAGEs) further contributes to endothelial cell dysfunction and vascular inflammation [72].

COPD-T2DM patients exhibit increased levels of oxidized low-density lipoproteins (ox-LDLs) and pro-inflammatory cytokines (TNF-α, IL-6, CRP), which trigger macrophage activation and foam cell formation, leading to plaque deposition in the arteries [73]. The persistent inflammatory state in COPD exacerbates vascular damage, increasing the likelihood of thrombotic events and cardiovascular instability [74].

Chronic systemic inflammation, oxidative stress, and endothelial dysfunction accelerate atherosclerosis progression, leading to arterial stiffness and heightened vascular resistance. Insulin resistance and hyperglycemia intensify these consequences by facilitating dyslipidemia, hypertension, and chronic vascular damage [75].

**Figure 2:**
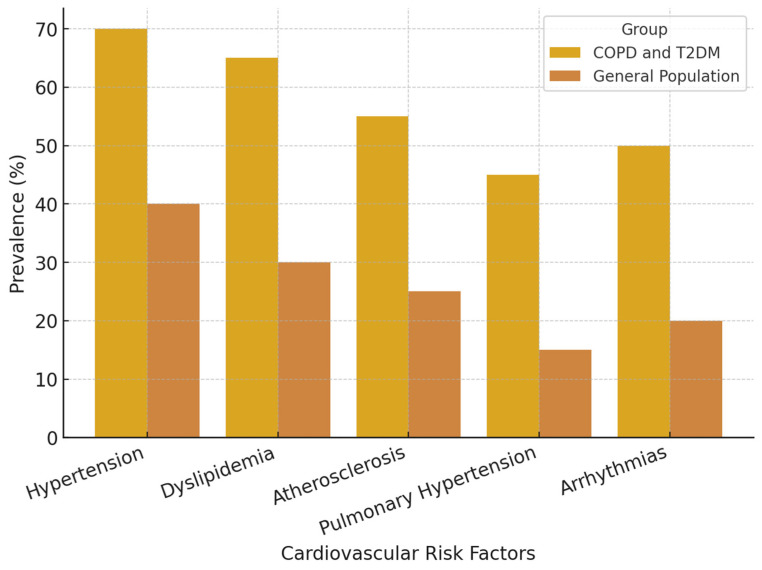
Comparison of cardiovascular risk factors in COPD and T2DM vs. the general population.

Patients with COPD and T2DM face an elevated risk of pulmonary hypertension due to chronic hypoxia, which induces vascular remodeling and right ventricular stress. This disorder may result in cor pulmonale and heightened mortality in those affected [69]. Moreover, autonomic dysfunction, frequently seen in both illnesses, can intensify cardiac problems, raising the risk of arrhythmias and sudden cardiac death (Figure 2).

Early diagnosis and vigorous management of cardiovascular disease risk factors are crucial to decreasing cardiovascular risk. Strategies must encompass rigorous glycemic regulation, the enhancement of lipid profiles, antihypertensive treatment, and the cessation of smoking. Pharmacological therapies, including statins, ACE inhibitors, and beta-blockers, should be contemplated, with meticulous selection processes to mitigate detrimental effects on pulmonary function [76]. Routine cardiovascular monitoring, including echocardiography and electrocardiographic evaluations, must be incorporated into the standard therapy for patients with COPD and T2DM to avert cardiovascular problems and enhance overall survival [77].

### 4.3. Impact on Quality of Life

Individuals with both COPD and T2DM suffer a notable decline in quality of life due to the convergence of symptoms including tiredness, dyspnea, and diminished activity capacity [78]. The interplay of pulmonary and metabolic dysfunction results in diminished physical activity, heightened psychological discomfort, and elevated incidences of depression and anxiety. The responsibility of managing several prescriptions, regular healthcare appointments, and dietary limitations exacerbates the difficulties encountered by individuals [79]. Implementing a comprehensive care approach that encompasses both physical and mental health is crucial for enhancing overall patient satisfaction and treatment adherence [80]. Patient education, mental health assistance, and organized illness management programs are essential to improve quality of life in this demographic [81].

### 4.4. Complications and Healthcare Utilization

The concomitant presence of COPD and T2DM results in heightened healthcare usage, characterized by higher hospitalizations, emergency department visits, and admissions to critical care units. Patients with comorbid diseases may need more-intricate treatment protocols, heightening the risk of polypharmacy and related adverse medication interactions. Furthermore, exacerbations of COPD in diabetic patients tend to be more severe, resulting in elevated in-hospital death rates and extended recovery durations [82]. Multidisciplinary care approaches that incorporate pulmonology, endocrinology, and general care are crucial for enhancing management and decreasing healthcare expenditures. Coordinated treatment techniques, telemedicine follow-ups, and proactive disease monitoring can enhance patient outcomes and decrease hospitalizations [83].

## 5. Pharmacological Considerations

### 5.1. Effects of COPD Treatments on Glucose Metabolism

The management of COPD often involves bronchodilators, inhaled corticosteroids (ICSs), and systemic corticosteroids, all of which may substantially affect glucose metabolism. Corticosteroids, especially systemic variants, are recognized for inducing hyperglycemia by enhancing hepatic gluconeogenesis, diminishing insulin sensitivity, and obstructing glucose absorption by peripheral organs [84]. Extended use of systemic corticosteroids correlates with an increased risk of steroid-induced diabetes, requiring vigilant monitoring of blood glucose levels in COPD patients with type 2 diabetes mellitus [85].

Inhaled corticosteroids (ICSs), while exhibiting a less systemic effect relative to oral corticosteroids, may still induce metabolic changes, particularly with prolonged usage. Research suggests that ICS may marginally elevate blood glucose levels, necessitating modifications in diabetes control protocols for COPD patients [9].

Systemic corticosteroids, such as prednisone and methylprednisolone, are associated with significant metabolic effects, including increased hepatic glucose production, reduced insulin sensitivity, and enhanced lipolysis, all of which contribute to worsening hyperglycemia and increased risk of diabetes onset or progression [86]. Chronic systemic steroid use has been linked to higher rates of steroid-induced diabetes, muscle catabolism, and central adiposity, all of which further deteriorate metabolic health in COPD-T2DM patients [24].

By contrast, inhaled corticosteroids (ICSs), commonly used in COPD management (e.g., fluticasone, budesonide), have a lower systemic bioavailability, thereby minimizing systemic metabolic impact [87]. However, studies suggest that high-dose ICS (>1000 μg/day of a fluticasone equivalent) is still associated with mild-to-moderate increases in blood glucose levels, particularly in patients already at risk of developing T2DM [48].

Given the metabolic risks of corticosteroid therapy, alternative COPD treatments should be considered in COPD-T2DM patients. These include the following:

Biologic Therapies: Monoclonal antibodies targeting the IL-5 (e.g., mepolizumab, benralizumab) or IL-4/IL-13 pathways (dupilumab) have shown efficacy in reducing airway inflammation and exacerbations while avoiding metabolic side effects. These therapies are particularly beneficial in COPD patients with eosinophilic phenotypes, providing a steroid-sparing approach [88].

Phosphodiesterase-4 (PDE-4) Inhibitors: Roflumilast, a PDE-4 inhibitor, has been shown to reduce airway inflammation and COPD exacerbations without adversely affecting glucose metabolism. PDE-4 inhibitors may be an effective alternative for patients at risk of corticosteroid-induced hyperglycemia [89].

Long-Acting Bronchodilators (LABAs and LAMAs): Long-acting β2-agonists (LABAs) and long-acting muscarinic antagonists (LAMAs) are first-line therapies in COPD and have no direct effects on glucose metabolism, making them safe for COPD-T2DM patients. Combination therapy with LABA/LAMA inhalers should be optimized before considering systemic steroids [90].

Macrolide Therapy: Chronic azithromycin therapy has shown anti-inflammatory effects in COPD and may serve as an adjunct therapy to reduce exacerbation frequency without the metabolic risks associated with corticosteroids [91].

Beta-agonists, often used as bronchodilators, may potentially affect glucose metabolism. Short-acting and long-acting beta-agonists (SABAs and LABAs) may increase insulin resistance and hepatic glucose synthesis via beta-adrenergic receptor activation [92]. Patients using these drugs must be consistently evaluated for exacerbating hyperglycemia, especially those with inadequately managed diabetes.

Considering these metabolic consequences, alternate therapeutic methods for COPD, such as the use of long-acting muscarinic antagonists (LAMAs) or minimizing systemic corticosteroid administration where possible, should be contemplated to alleviate detrimental metabolic effects [93].

### 5.2. Effects of Antidiabetic Medications on Pulmonary Function

Various antidiabetic medicines may influence pulmonary function, either beneficially or detrimentally, in individuals with COPD. Metformin, the primary medication for type 2 diabetes mellitus, has shown anti-inflammatory properties that may aid chronic obstructive pulmonary disease patients by diminishing systemic inflammation and oxidative stress [94]. Research indicates that metformin use correlates with enhanced pulmonary function and reduced exacerbations of COPD.

Glucagon-like peptide-1 receptor agonists (GLP-1 RAs) have surfaced as a potential category of therapeutics for individuals with both COPD and T2DM. GLP-1 receptor agonists, like liraglutide and semaglutide, have anti-inflammatory characteristics that may alleviate pulmonary inflammation [95]. Furthermore, they facilitate weight reduction, perhaps mitigating the respiratory strain associated with COPD in obese persons.

On the other hand, thiazolidinediones (TZDs), including pioglitazone, are associated with fluid retention, potentially aggravating pulmonary congestion and worsening respiratory symptoms in COPD patients. Consequently, TZDs are often excluded from this population [96].

Sodium–glucose cotransporter-2 inhibitors (SGLT2-Is) provide cardiovascular and renal protection; nonetheless, they need vigilant monitoring in COPD patients due to the possible dangers of dehydration and electrolyte imbalance, which may adversely affect respiratory performance [97]. For COPD patients with T2DM, tailored treatment strategies that account for the effects of antidiabetic medications on respiratory health are essential [63]. It is advisable for pulmonologists and endocrinologists to collaborate in order to enhance treatment results and reduce dangers.

### 5.3. Drug Interactions and Considerations in Comorbid COPD and T2DM

Considering the complexity of treating individuals with both COPD and T2DM, comprehending possible medication interactions is essential. Corticosteroids, often used for COPD, may disrupt glucose regulation, requiring enhanced blood sugar monitoring in diabetic individuals [85]. Prolonged use may need modifications in insulin or oral hypoglycemic medications to avert hyperglycemic emergencies.

Metformin is a first-line treatment for T2DM due to its ability to improve insulin sensitivity, reduce hepatic glucose production, and enhance glucose uptake in peripheral tissues. However, its use in COPD patients, particularly those with moderate-to-severe hypoxia, raises concerns regarding lactic acidosis. Metformin inhibits mitochondrial respiratory complex I, leading to reduced hepatic gluconeogenesis and increased anaerobic metabolism, which may result in lactate accumulation. In COPD patients with chronic hypoxia, reduced oxygen availability can further impair lactate clearance, thereby increasing the risk of metformin-associated lactic acidosis (MALA). COPD patients with severe respiratory insufficiency (PaO_2_ < 60 mmHg or those requiring long-term oxygen therapy) should be closely monitored when using metformin, with periodic lactate level assessments and considerations for alternative diabetes treatments in high-risk cases.

Systemic beta-agonists may induce tachycardia and elevate insulin resistance, requiring vigilance in individuals with cardiovascular comorbidities. In contrast, non-selective beta-blockers, often used for cardiovascular conditions in diabetic individuals, may trigger bronchospasms, exacerbating COPD symptoms. In certain instances, selective beta-blockers like metoprolol may be favored [98].

Non-selective beta-blockers (e.g., propranolol, nadolol) block both β_1_ (cardiac) and β_2_ (pulmonary) receptors, leading to airway smooth muscle constriction and reduced bronchodilation. These agents should be avoided in COPD patients due to their high risk of worsening respiratory symptoms [99].

Cardioselective beta-blockers (e.g., bisoprolol, metoprolol, nebivolol) selectively target β_1_ receptors in the heart with minimal β_2_ blockage, making them safer for COPD patients. Nebivolol, in particular, has additional vasodilatory and antioxidant properties that may improve endothelial function while minimizing pulmonary side effects [100].

Beta-blockers should not be withheld in COPD patients with a clear cardiovascular indication (e.g., post-myocardial infarction or heart failure), as the benefits often outweigh the risk of bronchospasm [101]. If beta-blocker therapy is required, cardioselective agents (e.g., bisoprolol, metoprolol succinate, or nebivolol) should be preferred over non-selective agents. Patients should be monitored for changes in lung function (FEV_1_ decline or worsening dyspnea) upon beta-blocker initiation [102]. Consideration of LAMAs (long-acting muscarinic antagonists) as a first-line therapy in COPD may counteract any potential beta-blocker-induced bronchoconstriction [103].

Diuretics, often taken for heart failure and hypertension in diabetics, may cause electrolyte imbalances, increasing the risk of dehydration and exacerbating COPD symptoms. Meticulous monitoring of potassium levels and hydration balance is essential [104].

Polypharmacy is a prevalent issue in people with COPD and T2DM. A multidisciplinary approach, including pulmonologists, endocrinologists, and primary care doctors, is crucial for optimizing pharmacological regimens and reducing adverse interactions [82,105]. Systematic drug evaluations and patient instruction of possible adverse effects may significantly enhance compliance and results.

## 6. Management Strategies

### 6.1. Multidisciplinary Approach

Managing patients with both COPD and T2DM requires a multidisciplinary strategy that includes pulmonologists, endocrinologists, cardiologists, dietitians, and primary care doctors. A coordinated approach is essential to tackle respiratory and metabolic dysfunctions while reducing risks from polypharmacy. Regular case discussions and collaborative treatment plans must be prioritized to guarantee thorough patient care [106].

### 6.2. Lifestyle Modifications

Smoking cessation: Smoking is a significant risk factor for both COPD and T2DM. Comprehensive smoking cessation programs, including behavioral treatment and pharmaceutical assistance (e.g., nicotine replacement therapy, varenicline), should be emphasized. Incorporating smoking cessation within standard healthcare appointments may significantly improve patient adherence [107].

Dietary modifications: A balanced diet abundant in whole grains, lean proteins, and healthy fats is crucial for glycemic regulation and inflammation reduction. Patients need to restrict their intake of processed meals, refined carbohydrates, and trans fats. Programs guided by dietitians, customized to meet patients’ metabolic and pulmonary requirements, may significantly enhance compliance with dietary guidelines [108].

Physical activity: Engagement in regular physical exercise may enhance insulin sensitivity and pulmonary function. Pulmonary rehabilitation programs must include strength training and aerobic activities customized to the patient’s breathing capability. Exercise programs should be created in collaboration with physiotherapists to guarantee that patients have safe and effective training routines [109].

### 6.3. Glycemic Control and Pulmonary Function Optimization

Monitoring Glycemic Control: Patients must have frequent HbA1c assessments and, if required, continuous glucose monitoring to maintain appropriate glycemic levels while avoiding the exacerbation of hypoxia-related problems. Insulin medication must be meticulously calibrated to prevent hypoglycemia, which might further impair respiratory function [110].

Pulmonary Function Testing: Spirometry and arterial blood gas analysis must be routinely performed to assess disease progression and therapy effectiveness. Detecting early deterioration in lung function may inform therapy modifications to mitigate disease development [111].

Vaccination: Annual influenza and pneumococcal vaccinations are advised to decrease the risk of respiratory illnesses. Patients need to have COVID-19 vaccines due to their heightened susceptibility to respiratory problems [112].

### 6.4. Pharmacological Optimization

Tailored COPD Treatment: Medications must be selected to reduce adverse metabolic effects. Whenever feasible, the use of ICS should be restricted, and long-acting bronchodilators should be prioritized. Customized treatment protocols must be created according to the severity of the illness and the patient’s reaction to therapy [113].

Diabetes Medications with Pulmonary Advantages: Metformin and GLP-1 receptor agonists may provide anti-inflammatory and cardiovascular advantages, making them favored options. Clinicians must maintain vigilance regarding the possibility of medication-induced problems and ensure that treatment regimens are appropriately tailored [95].

Cardiovascular Risk Management: ACE inhibitors, ARBs, and statins should be used when indicated to mitigate cardiovascular problems. Effectively managing hypertension and cholesterol levels may diminish the overall illness burden in these people [114].

### 6.5. Patient Education and Self-Management

Empowering Patients: It is essential to provide patients with educational tools on COPD-T2DM comorbidities, medication compliance, and lifestyle alterations. Digital and printed instructional resources, together with group seminars, may improve patient comprehension and adherence [115].

Telemedicine and Remote Monitoring: Digital health instruments, such as remote glucose monitoring and virtual pulmonary consultations, may augment disease care and boost patient outcomes. Mobile health apps that monitor respiratory and metabolic characteristics may assist patients and clinicians in identifying early indicators of illness aggravation [116].

Prognostic Awareness and Shared Decision-Making: Effective management of COPD and T2DM extends beyond pharmacological interventions to include comprehensive patient-centered discussions that enhance prognostic awareness (PA) and facilitate shared decision-making (SDM). Patients with both conditions often face progressive disease trajectories, frequent hospitalizations, and increased mortality risk, making early, structured conversations about prognosis essential.

PA refers to a patient’s understanding of their illness trajectory, treatment options, and anticipated outcomes, which has been shown to improve treatment adherence, psychological well-being, and quality of life. Studies indicate that prognostic discussions between clinicians, patients, and caregivers foster greater patient engagement, reduce anxiety, and support advanced care planning [117].

SDM is particularly relevant in COPD-T2DM care, where treatment strategies must be tailored to individual patient needs, preferences, and disease severity. Engaging family members and caregivers in decision-making processes ensures that treatment goals align with patient values, promoting continuity of care and better symptom management. Regular, iterative conversations about prognosis allow for timely adjustments to therapeutic strategies, ensuring that patients receive appropriate interventions at different stages of their disease [118].

Addressing Moral and Spiritual Distress: Chronic diseases such as COPD and T2DM not only impose significant physical burdens but also contribute to psychosocial and existential distress. Many patients experience spiritual distress, characterized by hopelessness, anxiety, loss of purpose, and fears about disease progression. Research indicates that unresolved spiritual distress can negatively impact treatment adherence, quality of life, and overall psychological well-being [119].

One critical yet often overlooked component of holistic disease management is addressing moral and spiritual concerns through integrated support systems. Patients with COPD-T2DM may struggle with questions regarding the meaning of suffering, uncertainty about prognosis, and ethical dilemmas surrounding life-prolonging treatments. Family involvement in these discussions can be instrumental in helping patients cope with disease burden, navigate complex medical decisions, and find comfort in shared decision-making [119].

By acknowledging the spiritual dimensions of chronic disease, healthcare providers can enhance patient resilience, reduce anxiety, and improve overall quality of life. Future research should explore the role of faith-based interventions, mindfulness practices, and ethical counseling in optimizing the psychosocial well-being of COPD-T2DM patients.

### 6.6. Addressing Psychological Well-Being

Mental Health Support: Individuals with COPD and T2DM often encounter anxiety and depression, which may impact disease management. Referral to counseling or psychiatric treatment needs to be considered. Cognitive behavioral treatment (CBT) has shown efficacy in enhancing adherence to medical regimens in patients with chronic illnesses [120].

Support Groups: Promoting engagement in COPD or diabetic support groups might enhance motivation and compliance with therapy. Peer support networks may alleviate feelings of loneliness and provide pragmatic strategies for illness management [121].

### 6.7. Long-Term Follow-Up and Risk Reduction

Scheduled Specialist Consultations: Regular follow-ups with pulmonologists, endocrinologists, and primary care physicians facilitate the modification of treatment regimens and the evaluation of long-term results. Periodic case evaluations should be undertaken to assess therapy effectiveness and patient welfare [122].

Preventive Health Strategies: Lifestyle advice, regular tests, and early treatments may reduce disease development and consequences. Incorporating preventative measures like weight control programs and consistent exercise into patient treatment regimens is essential for enhancing long-term health outcomes [123].

## 7. Discussion

The simultaneous development of COPD and T2DM poses a considerable therapeutic challenge owing to their overlapping pathophysiological pathways and the influence each illness exerts on disease progression and overall patient outcomes. This study has emphasized the complex interconnections among systemic inflammation, metabolic dysregulation, and pharmaceutical interactions that hinder the treatment of patients with both disorders [106,124,125]. Confronting these issues necessitates a holistic, multidisciplinary strategy that combines pulmonary and metabolic care, emphasizing individualized treatment plans customized to each patient’s need [126].

Effective treatment must focus on lifestyle improvements, including smoking cessation, dietary changes, and organized exercise regimens, to reduce disease development. Pharmacological techniques must be refined to guarantee that COPD medicines do not worsen glycemic management and that diabetes therapies do not impair lung function [127]. Consistent assessment of glucose levels and pulmonary function is crucial to avert acute exacerbations and chronic consequences.

In addition to pharmaceutical and lifestyle therapies, patient education and involvement are essential for fostering self-management and adherence to treatment regimens. Digital health techniques, including telemedicine and remote monitoring, provide effective options for enhancing illness management and facilitating early diagnosis of exacerbations [128]. Moreover, mental health assistance and involvement in peer support groups might augment psychological well-being and elevate patient motivation [129].

Future research should concentrate on creating customized treatments that simultaneously address COPD and T2DM. Research on innovative anti-inflammatory drugs, metabolic modulators, and precision medicine strategies will be essential in enhancing therapy alternatives [130]. Extensive clinical studies examining the long-term effects of integrated treatment techniques will provide significant insights into enhancing care and reducing the healthcare expenses linked to these comorbid illnesses [131].

Collaboration between healthcare providers, researchers, and policymakers is essential to establish effective treatment guidelines and ensure a holistic approach to managing COPD and T2DM. By fostering innovation and evidence-based practice, a more comprehensive and patient-centered framework can be developed to enhance quality of life, reduce disease burden, and improve overall survival rates in affected individuals [132]. The interplay between COPD and T2DM presents significant clinical challenges that necessitate an integrated and multidisciplinary approach. Understanding the shared pathophysiological mechanisms and optimizing pharmacological and non-pharmacological management strategies are essential for improving patient outcomes [133].

## 8. Future Perspectives

### 8.1. Advances in Research and Treatment

The future therapy of COPD and T2DM depends on the development of innovative pharmaceutical strategies that target the same pathophysiological pathways underlying both diseases. Progress in precision medicine, tailored anti-inflammatory treatments, and metabolic modulators may result in more-efficacious therapeutic alternatives. The use of artificial intelligence (AI) and machine learning in the form of illness prediction models may facilitate early identification and individualized treatment planning.

Recent studies on the gut microbiome and its contribution to chronic inflammation indicate that microbiota-targeted therapy, such as probiotics and dietary modifications, may be effective in controlling both COPD and T2DM. Furthermore, genetic and biomarker studies may provide insights into patient-specific disease pathways, allowing highly personalized therapy protocols that enhance results and reduce side effects.

### 8.2. Role of Digital Health Technologies

The increasing popularity of digital health technologies, such as telemedicine, wearable sensors, and mobile health apps, offers novel prospects for remote monitoring and patient interaction. These advances may enhance treatment adherence, enable early intervention, and alleviate the strain on healthcare systems by decreasing hospital admissions and emergency visits.

Decision support technologies driven by artificial intelligence might assist healthcare practitioners in making improved treatment choices, optimizing prescription regimens, and detecting patients at elevated risk of exacerbation. Furthermore, the use of remote pulmonary function testing and real-time glucose monitoring has the potential to transform disease management by facilitating ongoing evaluation of health status outside clinical environments.

### 8.3. Preventive Strategies and Public Health Initiatives

Priority should be given to preventive interventions designed to diminish the incidence and severity of COPD and T2DM. Public health campaigns that advocate for smoking cessation, promote physical exercise, and educate consumers on nutritious eating practices may substantially reduce disease burden. Government and healthcare entities must cooperate to develop policies that promote preventative healthcare and early intervention initiatives.

Workplace wellness programs, community screening campaigns, and tailored treatments for high-risk groups might significantly alleviate the burden of these illnesses. Moreover, augmenting financial support for research on the socioeconomic underpinnings of COPD and T2DM would enable policymakers to design more-effective preventative programs that tackle gaps in healthcare access and disease outcomes.

### 8.4. The Need for Longitudinal Studies

Longitudinal research evaluating the effects of combined COPD-T2DM treatment regimens are essential to providing evidence-based recommendations. Future clinical studies should investigate the long-term effects of innovative pharmacological drugs and lifestyle modifications to assess their effectiveness in enhancing patient outcomes. Moreover, genetic and biomarker studies may provide additional insights into the personalized management of individuals with COPD and T2DM.

International collaborative research initiatives, including extensive multicenter trials, are essential for comprehending the connections between COPD and T2DM across varied populations. By collecting longitudinal data on illness development, treatment responses, and quality of life indicators, researchers may formulate customized strategies that more effectively meet patient requirements. Moreover, future research should integrate real-world evidence studies and patient-reported outcome measures to accurately reflect the lived experiences of people treating both illnesses.

## Data Availability

Data are contained within the article.

## Appendix A

### Appendix A.1

**Table A1:** Literature search strategy and keywords used.

Database	Search Terms Used	Filters Applied	Number of Results
PubMed	“COPD AND Type 2 Diabetes” OR “Chronic Obstructive Pulmonary Disease AND Metabolic Dysfunction”	2010–2025, English, Humans	324
Scopus	“COPD AND Insulin Resistance” OR “Lung Function AND Diabetes”	Peer-reviewed journals, last 15 years	198
Web of Science	“Systemic Inflammation AND COPD AND Diabetes”	Clinical studies, meta-analyses only	156

### Appendix A.2

**Table A2:** Inclusion and exclusion criteria for study selection.

Criteria	Inclusion	Exclusion
Population	Adults diagnosed with COPD and T2DM	Pediatric populations, animal studies
Study Type	Clinical trials, observational studies, meta-analyses	Editorials, commentaries, case reports
Language	English	Non-English
Publication Year	2010–2025	Before 2010
Study Quality	Assessed using SANRA, MMAT	Low methodological rigor

### Appendix A.3

**Table A3:** Quality assessment scores using SANRA, MMAT.

Study	SANRA Score (0–12)	MMAT Score (0–100%)
Study A	10	85%
Study B	9	80%
Study C	8	75%
Study D	7	70%
Study E	8	75%
Study F	10	85%
Study G	9	80%
Study H	9	80%
Study I	8	75%
Study J	9	80%
